# Separation of the bacterial species, *Escherichia coli*, from mixed-species microbial communities for transcriptome analysis

**DOI:** 10.1186/1471-2180-11-59

**Published:** 2011-03-22

**Authors:** Dongjuan Dai, Diane Holder, Lutgarde Raskin, Chuanwu Xi

**Affiliations:** 1 Department of Environmental Health Sciences, University of Michigan, Ann Arbor, MI, USA, 48109, USA; 2 Department of Civil and Environmental Engineering, University of Michigan, Ann Arbor, MI, USA; 3 Proficiency and Validation Services Section, USDA, APHIS, FADDL, Greenport, NY, USA

## Abstract

**Background:**

The study of bacterial species interactions in a mixed-species community can be facilitated by transcriptome analysis of one species in the community using cDNA microarray technology. However, current applications of microarrays are mostly limited to single species studies. The purpose of this study is to develop a method to separate one species, *Escherichia coli *as an example, from mixed-species communities for transcriptome analysis.

**Results:**

*E. coli *cells were separated from a dual-species (*E. coli *and *Stenotrophomonas maltophilia*) community using immuno-magnetic separation (IMS). High recovery rates of *E. coli *were achieved. The purity of *E. coli *cells was as high as 95.0% separated from suspended mixtures consisting of 1.1 - 71.3% *E. coli*, and as high as 96.0% separated from biofilms with 8.1% *E. coli *cells. Biofilms were pre-dispersed into single-cell suspensions. The reagent RNA*later *(Ambion, Austin, TX) was used during biofilm dispersion and IMS to preserve the transcriptome of *E. coli*. A microarray study and quantitative PCR confirmed that very few *E. coli *genes (only about eight out of 4,289 ORFs) exhibited a significant change in expression during dispersion and separation, indicating that transcriptional profiles of *E. coli *were well preserved.

**Conclusions:**

A method based on immuno-magnetic separation (IMS) and application of RNA*later *was developed to separate a bacterial species, *E. coli *as an example, from mixed-species communities while preserving its transcriptome. The method combined with cDNA microarray analysis should be very useful to study species interactions in mixed-species communities.

## Background

Microorganisms in natural environments rarely grow as single species, but grow as mixed species consortia in which a variety of intra- and inter-species interactions take place [1,2]. Previous studies have shown that species interactions play an important role in the development, composition, structure and function of microbial consortia in biofilms as well as in suspended growth communities [3-5]. Studies of species interactions have promoted the understanding of microbial activities in mixed-species communities [6-8].

Identification of relevant genes is an important step toward the elucidation of the molecular mechanisms of species communication. cDNA microarray technology has been widely used for mono-species cultures, but only a few cDNA microarray studies have been performed for mixed-species consortia due to broad cross hybridization among species [6,9,10]. Variable conservation of genes existed across bacterial species [11]. Non-target transcripts have been shown to cross hybridize in oligonucleotide microarray studies [12]. The problem was addressed previously by carefully selecting co-cultures consisting of one gram-negative and one gram-positive strain, so that RNA could be selectively extracted from one strain [6,9]. However, for most mixed-species communities, selective RNA extraction is not possible and a method needs to be developed in order to apply cDNA microarray technology to such communities.

Separating the target species from other community members before extracting RNA could be an approach in minimizing cross hybridization on microarrays. Immuno-magnetic separation (IMS) using magnetic force to recover target cells with paramagnetic beads and specific antibodies has been widely used [13-15]. The IMS procedure has been standardized [16]. However, isolated cells have not been considered for cDNA microarray analysis.

While the purity of recovered cells is important for microarray analysis, it was not always considered in previous studies. In addition, preserving the transcription profile of target cells during IMS is critical for downstream microarray analysis and is the most important concern addressed in this study. RNA*later *(Ambion, Austin, TX) has been used to stabilize and protect cellular RNA during sample storage. However, the effect of RNA*later *on IMS separation efficiency has not been explored previously.

This study tested and developed a method that can be used to study the transcriptome of one species in mixed-species communities, including suspended and biofilm communities. *Escherichia coli *was selected as the target species in this study and *Stenotrophomonas maltophilia *as a background species, because we are interested in the interactions between these two species when *E. coli *forms biofilms in drinking water distribution systems*. E. coli *is an important indicator of fecal contamination and is detected in some water distribution systems [17]. *S. maltophilia *is a ubiquitous species in water systems. For example, the abundance of *Stenotrophomonas *spp. was 2-6% in a pilot drinking water distribution system [18]. Isolation of both *E. coli *and *S. maltophilia *from water filtration and distribution systems [19] suggests that they share the same niches in engineered systems and that interactions between them take place in such systems.

The efficiency of IMS to separate *E. coli *from various suspended mixtures and biofilms consisting of *E. coli *and *S. maltophilia *was evaluated in this study. The recovery and purity of separated *E. coli *cells were reported. Changes in the transcription profiles of *E. coli *cells due to sample processing and cell separation were quantified by cDNA microarray analysis and quantitative PCR (qPCR) to evaluate the effectiveness of the developed method. We also discussed that the method could be applied to study other species of interest in mixed community systems and was not limited to the example species used in this study as long as a specific antibody for the target species is available.

## Results and Discussion

### Recovery rate of *E. coli*

**Figure 1:**
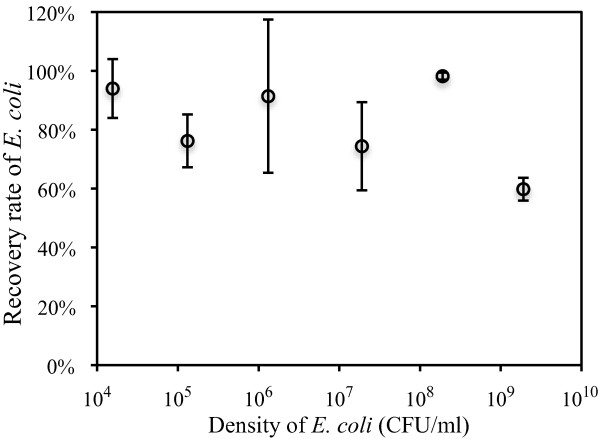
**Recovery rates of *E. coli *cells after immuno-magnetic separation**. Recovery rates of *E. coli *cells after one-step IMS from suspensions of *E. coli *with densities adjusted from approximately 10^4 ^to 10^9 ^CFU/ml. Error bars indicate standard deviations of triplicate plate counts.

The recovery rate of *E. coli *by immuno-magnetic separation (IMS) from a series of suspended cultures was determined first. A general antibody of *E. coli *(polyclonal anti-*E. coli *antibody (ViroStat, Portland, ME)) was used in this study. Using this antibody, the recovery rate of *E. coli *was 74.4-98.2% when separated from suspended cultures with a density up to 1.9 × 10^8 ^CFU/ml (Figure 1). However, the recovery rate dropped to 59.8% for samples with ten-fold higher cells (1.9 × 10^9 ^CFU/ml), which may have exceeded the capacity of separation columns used in IMS (Figure 1). Therefore, *E. coli *cell densities in samples were adjusted to less than 2 × 10^8 ^CFU/ml for subsequent IMS.

Determining the recovery rate of target species is important when IMS is used to separate target species for subsequent cDNA microarray analysis. High recovery rates yield sufficient cells for RNA extraction, especially for low-abundance target species or when limited sample amounts are available. High recovery rates of *E. coli *were achieved from samples with a wide range of cell densities (10^4^-10^8 ^CFU/ml). The recovery rates observed in this study were generally higher than those reported previously (53-82%) [20-22].

### Purity of *E. coli *separated from dual-species cultures

**Figure 2:**
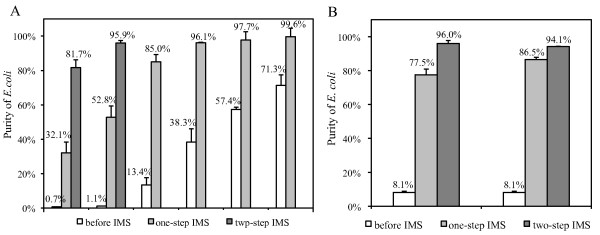
**Purity of *E. coli *cells before and after separation from suspended mixtures and biofilms**. Purity of *E. coli *cells before and after one- or two-step IMS from (A) suspended mixtures and (B) biofilms of *E. coli *and *S. maltophilia *cells. Suspended mixtures were prepared by mixing suspended *E. coli *cells (10^4^-10^6 ^CFU/ml) with *S. maltophilia *cells (10^5^-10^8 ^CFU/ml). Biofilms were scraped from a flow-cell system and dispersed into suspensions of single cells (*E. coli *2.3 × 10^6 ^CFU/ml, *S. maltophilia *2.6 × 10^7 ^CFU/ml). Two independent IMS experiments were performed for aliquots of dispersed biofilms. Error bars indicate standard deviations of two or three replicate plate counts.

Suspended mixtures containing 0.7-71.3% *E. coli *cells (10^4^-10^6 ^CFU/ml *E. coli *and 10^5^-10^8 ^CFU/ml *S. maltophilia*) were used to evaluate IMS for separating and purifying *E. coli *cells from various communities. One-step IMS enriched *E. coli *cells to a purity of over 95% from mixtures with 38.3-71.3% *E. coli *cells (Figure 2A). But the purity of *E. coli *cells after one-step IMS was too low to be acceptable (32.1-52.8%) when separated from mixtures containing less *E. coli *cells (0.7-13.4%) (Figure 2A). Therefore, a second IMS was performed and *E. coli *cells were successfully enriched to a high purity of 95.9% from mixtures containing as little as 1.1% *E. coli *cells (Figure 2A).

Previous studies did not report whether other species, such as *S. maltophilia*, would bind to the anti-*E. coli *antibody [21-23]. The high purity of *E. coli *obtained by one- or two-step IMS (> 95%) (Figure 2A) suggested that cross-reactivity, if there was any, was not a concern. Low purity of *E. coli *(32.1-52.8%) obtained from mixtures with small percentages of *E. coli *(0.7-13.4%) was a result of a small fraction (1%) of *S. maltophilia *cells accumulation in the LS columns, in which magnetically labeled *E. coli *cells were held during washing. When *S. maltophilia *was dominant in samples (e.g., *S. maltophilia *> 90% and *E. coli *< 10%), the relatively low accumulation of *S. maltophilia *(1%) yielded high number of *S. maltophilia *cells in absolute terms, resulting in low purity of *E. coli *after IMS. However, since the accumulated *S. maltophilia *cells were not actually bound to the anti-*E. coli *antibody, they were removed during the second IMS, resulting in highly purified *E. coli *cells (Figure 2A).

Real dual-species biofilms harvested from flow cell systems were used to investigate whether IMS could also separate *E. coli *from biofilms. The biofilm matrix was homogenized to disperse cell aggregates into a suspension of single cells before IMS. Two independent separations were performed for aliquots of dispersed biofilms. Two-step IMS was able to enrich *E. coli *to around 95% from biofilms containing only 8.1% *E. coli *(2.3 × 10^6 ^CFU/ml *E. coli *and 2.6 × 10^7 ^CFU/ml *S. maltophilia*) (Figure 2B). The results demonstrated the feasibility of using IMS to separate *E. coli *cells from biofilms.

It is important to obtain target cells in high purity from mixed species communities for subsequent cDNA microarray analysis in order to effectively limit cross hybridization. The results showed that a high purity of *E. coli *cells could be obtained by IMS from different mixed-species communities (suspensions or biofilms) with various amounts of *E. coli *cells (0.7-71.3%).

### Preservation of RNA integrity during cell separation

Preserving RNA integrity during IMS is critical when collected cells are used for subsequent cDNA microarray analysis. RNA*later *(Ambion, Austin, TX) has been used widely to preserve RNA in bacterial cells, but the impact of RNA*later *on IMS performance was unknown. The recovery rate of *E. coli *dropped to 1% if cells remained in RNA*later *during the complete IMS procedure. This may be the result of antibody denaturing by the global protein denaturing reagents present in RNA*later*. Alternative products, such as RNAprotect (Qiagen, Germantown, MD), contain similar denaturing reagents and are expected to show similarly reduced recoveries.

In order to overcome this problem, RNA*later *was removed during some steps of the IMS procedure. Samples were stored in RNA*later *at 4°C overnight to allow the reagent to penetrate into bacterial cells and to stabilize intracellular RNA. RNA*later *was then removed and bacterial cells were resuspended in separation buffer just before incubation with antibody and microbeads. One-step IMS enriched *E. coli *to a similar level as shown in Figure 2A and removed over 99% of *S. maltophilia *cells (data not shown). The results confirmed that the modified protocol did not affect the recovery and purity of *E. coli *processed by IMS.

Pre-stabilization in RNA*later*, quick sample processing (~30 min), low working temperature (4°C), and maintaining an RNAase-free environment were combined to limit RNA degradation during IMS, since RNA*later *had to be removed during some steps of the IMS procedure. The effectiveness of these strategies in preserving the integrity of RNA was confirmed by observing, using agarose gel electrophoresis, high quality RNA extracted from cells treated with the IMS procedure (data not shown).

### Impact of cell separation on *E. coli *transcription profiles

To evaluate whether gene expression profiles were changed during sample processing (biofilm dispersion) and IMS cell sorting, cDNA microarray analysis was used to compare gene expressions of *E. coli *cells without dispersion and IMS (unsorted cells) and with dispersion and IMS (sorted cells). To eliminate the possible impact of any non-target RNA (from the small amount (< 5%) of *S. maltophilia *cells remaining in enriched collections), pure cultures of *E. coli *rather than dual-species mixtures were used to study changes in transcription profile of *E. coli *due to cell separation. To this end, pure cultures of *E. coli *were processed using the same procedure used for dual-species biofilm treatment, including cell dispersion and IMS.

**Figure 3:**
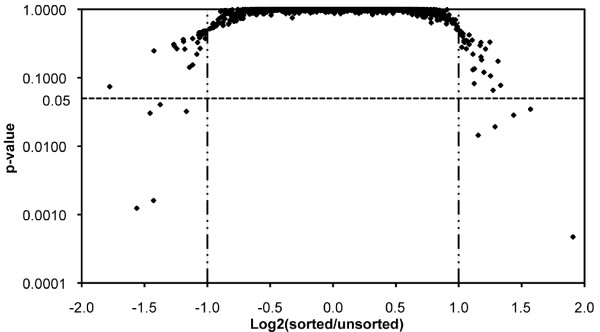
**Plot of gene expression of sorted/unsorted cells**. Plot of one-sample T-test p-values with fold-change in gene expression for all ORFs in microarray study I. Vertical lines show the cutoff of fold-change of 2 (Log_2 _ratio of ± 1), while the horizontal line shows the cutoff of p-value 0.05. Genes located in the left-bottom corner (Log_2 _ratio <-1 and p-value <0.05) and in the right-bottom corner (Log_2 _ratio >1 and p-value <0.05) were considered to have their expressions changed due to dispersion/homogenization and IMS (immuno-magnetic separation) cell sorting. A total of ten genes were selected using these criteria, eight of which also differentially expressed in the independent microarray study II.

Differentially expressed genes were identified based on fold-change and statistical significance compared to the control (Figure 3) [24]. Only 10 and 45 of the 4,289 ORFs exhibited differential expression in two independent microarray studies I and II, respectively (each microarray study was performed with two technical replicates of microarray slides and each microarray slide had three built-in replicates). A complete list of the differentially expressed genes is provided in Additional File 1: Full list of genes differentially expressed in sorted *E. coli *cells. Only eight of these genes showed consistent changes in both of the independent microarray studies (Table 1), with three genes up-regulated and five genes down-regulated in sorted *E. coli *cells in comparison to unsorted *E. coli *cells. The fold-change of gene expression ranged from 2.7 to -4.6 (Table 1). Differential expression of the eight genes in sorted and unsorted *E. coli *cells, as identified by the cDNA microarray analysis, was verified with qPCR using the 16S rRNA gene as a housekeeping gene. Seven out of the eight genes showed the same trend of differential expression (up-regulated or down-regulated in sorted cells) as revealed by the cDNA microarray analysis (Table 1). Moreover, the qPCR results indicated that five out of the eight genes exhibited less than two-fold change in sorted/unsorted cells. It suggested that the actual number of genes affected by the performance of IMS sorting may be even less than eight. It further confirmed the effectiveness in preserving the transcriptome of *E. coli *cells by the method developed in this study.

**Table 1:** Genes identified as differentially expressed^# ^between IMS sorted *E. coli *cells versus unsorted *E. coli *cells* by the method of cDNA microarray and their differential expression confirmed with another method of qPCR

		Fold-change of expression (sorted/unsorted)			
Gene	Locus Tag			qPCR (sorted/unsorted)	Annotation^⊕^
		Microarray study I	Microarray study II		
*tldD*	b3244	2.7 ± 1.4^Ψ^	2.7 ± 1.4	1.1 (0.8, 1.48)^&^	Predicted peptidase
*proW*	b2678	2.4 ± 1.1	3.3 ± 1.3	-1.6 (-1.1, -2.3)	Glycine betaine transporter subunit
*ansP*	b1453	2.2 ± 1.1	2.5 ± 1.1	1.2 (0.9, 1.48)	*L*-asparagine transporter

*ydhB*	b1659	-2.2 ± 1.1	-2.9 ± 1.2	-5.0 (-4.4, -5.7)	Predicted DNA-binding transcriptional regulator
*yhhN*	b3468	-2.6 ± 1.3	-3.1 ± 1.2	-3.1 (-2.8, -3.4)	Conserved inner membrane protein
*ygeV*	b2869	-2.7 ± 1.1	-3.3 ± 1.4	-1.6 (-1.4, -1.7)	Predicted DNA-binding transcriptional regulator
*flhE*	b1878	-2.7 ± 1.2	-3.2 ± 1.2	-1.8 (-1.7, -2.0)	Conserved protein
*yicG*	b3646	-3.0 ± 1.2	-4.6 ± 1.3	-3.7 (-3.3, -4.1)	Conserved inner membrane protein

**^#^**Fold-changes of gene expression were significantly different from 2, with one-tail t-tests performed (p < 0.05).

*Sorted *E. coli *cells: *E. coli *cells treated with dispersion/homogenization and IMS cell sorting after pre-stored in RNA*later*; Unsorted *E. coli *cells: *E. coli *cells continuously stored in RNA*later *without any treatment.

^⊕^Annotations are updated according to records of *E. coli *K-12 MG1655 in NCBI Entrenz Gene Database.

^Ψ^Mean ± geometric standard deviation from two replicate slides, with three built-in replicates in each slide; positive and negative values indicate up- and down-regulation, respectively, in dispersed and IMS sorted cells. Geometric standard deviation is 2^SD^, where SD is standard deviation of log_2 _transformation of fold-change.

^&^Mean of the fold change in gene expression from four replicates (ranges of fold change are given in parentheses), positive and negative values indicate up- and down- regulation, respectively, in dispersed and IMS sorted cells quantified by the method of qPCR.

This study developed and evaluated a method that can be used to study the transcriptome of one species in mixed-species communities, including suspended cultures and biofilms. It was not surprising to find some genes with changed expression after several treatment steps, i.e., cell homogenization/dispersion, re-suspension in buffer, and IMS cell sorting. However, the number of differentially expressed genes was very low (eight genes correspond to 0.2% of the 4,289 ORFs). We further searched in the literature whether the eight differentially expressed genes were involved in species interactions or biofilm formation, since this method was specifically developed to identify genes involved in bacterial species interactions in mixed-species communities, including in biofilm communities. None of the eight genes has been shown to be involved in bacterial species interactions. With regard to biofilm formation, only one of the eight genes, *flhE*, showed a potential effect on biofilm formation by *Salmonella typhimurium *in one study [25]. Thus, it can be concluded that transcription profiles of enriched *E. coli *cells were well preserved during IMS and the use of IMS to separate *E. coli *showed no obvious adverse effects for future applications of this method to study species interactions, including in biofilms.

## Conclusions

Good recovery, high purity and preserved transcription profiles of *E. coli*, which was used as an example species, indicate that the method developed in this study can be used to study transcription profiles of *E. coli *in a mixed community with *S. maltophilia*. Although *S. maltophilia *was used as the background species in this study, this method can be used to remove other background species that exhibit little cross binding with the antibody used, even if the background species would be phylogenetically closer to *E. coli *than *S. maltophilia*. Similarly high recoveries and purities of *E. coli *were achieved when sorted from mixtures of *E. coli *and a *Salmonella *species (Dr. Matthew Chapman, personal communication). In addition, the method should not be limited to studies of *E. coli*, and it can be applied to study other species of interest for which specific antibodies are available. While antibody dosage and homogenization intensity need to be determined when separating other species of interest, the basics of the method presented here can be applied to other communities. The applicability of the method to study real mixed-species communities has been tested by our recent study in identifying genes of *E. coli *involved in interactions with *S. maltophilia *(manuscript in preparation). Gene identification of species interactions can lead to further our understanding of mechanisms of species interactions as shown by previous studies [9]. The method developed here thus has the potential to contribute to studies in which understanding the mechanisms of species interactions is an important component.

## Methods

### Bacterial strains and suspended mixtures

Overnight cultures of *E. coli *K-12 PHL644/pMP4655 (carrying a *gfp *gene under the control of a constitutive promoter) and *S. maltophilia*/pBPF-mCherry were grown in Luria-Bertani (LB) broth supplemented with tetracycline (80 μg/ml) or gentamicin (20 μg/ml) at 34°C with continuous shaking (200 rpm). Cells were pelleted by centrifugation (3,300 × g, 4°C, 3 min), re-suspended, and diluted in 1× phosphate buffered saline (PBS, pH 7.4) supplied with 0.5% bovine serum albumin (BSA) (Pierce, Rockford, IL). A series of artificial mixtures of *E. coli *and *S. maltophilia *were prepared by mixing the PBS re-suspended and diluted *E. coli *and *S. maltophilia *cells at different ratios.

Biofilms were cultivated on the inner surface of silicon tubing (Cole-Parmer, Vernon Hills, IL) in flow cell systems as described previously [26]. Briefly, a flow cell system was assembled, sterilized, and conditioned by running 0.1× LB broth (10-fold diluted LB broth, 1 ml/min) at room temperature (20-25°C). Operation was paused for one hour to allow inoculation with *S. maltophilia *and *E. coli *mixed at a ratio of 1:1. After three days of growth, biofilms were scraped into 1× PBS and pre-homogenized on ice using a homogenizer (OMNI TH, Marietta, GA) set at the lowest speed for 30 seconds. Biofilms were further dispersed into single cells using the same homogenizer set at the maximum speed for two minutes. Over 99% of bacterial cells in the biofilm matrix were dispersed into single cells. The dispersed biofilm cells were then diluted in 1× PBS (with 0.5% BSA) for IMS.

### Immuno-magnetic separation

One milliliter of samples was incubated with 10 μl anti-*E. coli *antibody (ViroStat, Portland, ME) for 10 min with gentle shaking. Bacterial cells were pelleted by centrifugation (3,300 × g, 4°C, 3 min) and re-suspended in 100 μl separating buffer (1× PBS, 0.5% BSA, 2 mM EDTA, pH 7.4) (EDTA: ethylenediaminetetraacetic acid). 10 μl streptavidin microbeads (Miltenyi Biotec, Auburn, CA) were added and incubated at 4°C in the dark for 10 min. Separation of *E. coli *cells was performed in LS columns and a midi MACS^® ^separator (Miltenyi Biotech, Auburn, CA) following the protocol provided by the manufacturer, except that one more washing step was added to remove more *S. maltophilia *cells. In a two-step IMS, enriched cells from the first step IMS were directly transferred into a new LS column for the second separation. Densities of *E. coli *and *S. maltophilia *cells in samples and IMS enriched collections were measured using a plate-counting method with selective agar. Cell densities were used to calculate recovery and purity of *E. coli *after IMS.

The protocol was amended with the use of RNA*later *when enriched cells were used for microarray study. Bacterial cells were re-suspended in RNA*later *rather than PBS after sample collection and kept at 4°C overnight, followed by homogenization. RNA*later *was removed and cells were re-suspended in separating buffer just before IMS. During column separation, the buffer was additionally supplied with 10% (v/v) RNA*later*. Enriched cells were immediately stored in RNA*later*. The whole procedure was performed at 4°C. All buffers, reagents, and pipette tips were nuclease-free and pre-cooled.

### Microarray study

Pure *E. coli *cultures were used to evaluate the effect of separation on the transcriptome by microarray analysis. Suspended *E. coli *cultures were harvested from an annular reactor (1320 LJ, BioSurface Technologies, Bozeman, MT), supplied with 0.1× LB broth (100 ml/h) for 7 days after inoculation. Aggregates were removed from broth cultures by filtration (5.0 μm Millipore, Billerica, MA). Suspended *E. coli *cells were immediately re-suspended in RNA*later *and stored at 4°C overnight. One aliquot of RNA*later *stored *E. coli *cells served as the control ("unsorted" cells) and was kept in RNA*later *without further treatment. The other aliquot was treated to acquire "sorted" cells as described above using the amended protocol. Samples collected independently from a second annular reactor served as a biological replicate for the microarray study.

RNA*later *was removed by filtration with a membrane (0.22 μm, Millipore, Billerica, MA) from *E. coli *cells just before RNA extraction for both "unsorted" and "sorted" cell collections. RNA extraction was based on a hot SDS/phenol protocol [27]. A step of bead beating (BioSpec, Bartlesville, OK) for one minute was added to break cells, and all phenol/chloroform/isoamyl alcohol washes were performed in phase lock gels (5 Prime, Fisher Scientific, Pittsburgh, PA). DNA was removed from extracted RNA with Turbo DNase treatment (Ambion, Austin, TX) at 37°C for 30 min followed by purification with an RNeasy Mini Kit (Qiagen, Germantown, MD). The quality of RNA was examined by gel electrophoresis using E-gel with SYBR Safer (Invitrogen, Carlsbad, CA). High quality RNA was further re-precipitated, concentrated, and stored at -80°C.

RNA was reverse transcribed into cDNA using random hexamers (pd(N)_6_) (GE Healthcare, Piscataway, NJ) and labeled with Amersham CyDye Post-Labeling Reactive Dye (Amersham Biosciences, Piscataway, NJ) following the protocol provided by the Amino Allyl cDNA Labeling Kit (Ambion, Austin, TX). The quantity and labeling efficiency of cDNA was measured using a NanoDrop Spectrophotometer (ND-1000, Thermo Scientific, Wilmington, DE).

Microarray slides for *E. coli *were purchased from the University of Alberta (Edmonton, AB, Canada). Each slide contained three replicates of 5,978 70-mer oligonucleotides representing three *E. coli *strains (4,289 of them were for *E. coli *K-12). Sample preparation and loading, slide prehybridization, hybridization and washing were performed according to Corning protocols (GAPS II coated slides, Corning Inc., Lowell, MA). An extended 4-h prehybridization using a higher BSA concentration (1 mg/ml) was found to perform best in reducing background noise. Hybridization was in a Corning Microarray Hybridization Chamber (Corning Inc.) in 42°C water bath.

Microarray slides were scanned with a Virtek ChipReader (Virtek Vision, Waterloo, ON, Canada). Spots on scanned images were recognized and pixel intensity for each spot was quantified using the TIGR software Spotfinder (v3.1.1). Gene expression data were analyzed in the software Acuity 4.0 (Molecular Devices, Sunnyvale, CA). LOWESS normalization was performed for every microarray with three iterations using a smoothing factor of 0.4. Hybridized spots with oligonucleotides for strain *E. coli *K-12 having a high QC (quality control) value (> 0.1), good flag tags (A, B and C) in both Cy3/Cy5 channels were chosen for further analysis. One sample t-tests were performed across replicates. Step-down Bonferroni-Holm was used for the correction of multiple hypotheses testing. Genes with at least two-fold change in expression (p-value < 0.05) were considered to have changed expression during sample dispersion and IMS. Microarray data were deposited in NCBI Gene Expression Omnibus database (GSE22885).

### Quantitative PCR (qPCR)

Primers for qPCR confirmation of the differential expression of eight identified genes in Table 1 are listed in Additional File 2: qPCR primers for nine tested genes. The primers were designed using the software Primer Premier (Palo Alto, CA) and synthesized by Integrated DNA Technologies (Coralville, IA). Annealing temperatures and qPCR efficiency were optimized with PCR products using *E. coli *genomic DNA as template. The 16S rRNA gene was selected as the housekeeping gene. The amplification efficiency for target genes was near 100% and within 5% of the housekeeping gene of 16S rRNA. Total RNA from sorted and unsorted *E. coli *cells were reverse transcribed to cDNA using a reverse transcription kit (Applied Biosystems, Carlsbad, CA). cDNA was diluted 10- and 100-fold and 1 μl was assembled for qPCR reactions using the SYBR Green PCR Master Mix (Applied Biosystems, Carlsbad, CA). Differential expression of the same gene in sorted and unsorted *E. coli *was calculated with the ΔΔCt method from four replicates. The PCR program included a cycle of 95°C for 10 min, 35 cycles of 30 seconds at 94°C, 30 seconds at the optimized annealing temperature for each set of specific primers and 30 seconds at 72°C, and a melting curve analysis from 60°C to 95°C at the end.

## Authors' contributions

DD carried out experimental studies and data analysis, participated in the design of the study, and drafted the manuscript. DH was involved in microarray data analysis and revising the manuscript. LR participated in the design of the study and revising the manuscript. CX conceived of the study, participated in its design and coordination, and revised the manuscript. All authors read and approved the final manuscript.

## Supplementary Material

Additional file 1 **Full list of genes differentially expressed in sorted *E. coli *cells**. Full list of genes of *E. coli *differentially expressed in IMS sorted *E. coli *cells versus unsorted *E. coli *cells in two independent microarray studies I and II. Click here for file

Additional file 2 **qPCR primers for nine tested genes**. List of primers and their optimized annealing temperatures used in qPCR to confirm differential expression in IMS sorted versus unsorted *E. coli *cells. Click here for file
