# Supplementary material for: Separation of the bacterial species, Escherichia coli, from mixed-species microbial communities for transcriptome analysis
Source: BMC Microbiol. 2011 Mar 22;11:59. doi: 10.1186/1471-2180-11-59 (PMC3076228; doi:10.1186/1471-2180-11-59)
Supplement: Additional file 1 — Full list of genes differentially expressed in sorted E. coli cells. Full list of genes of E. coli differentially expressed in IMS sorted E. coli cells versus unsorted E. coli cells in two independent microarray studies I and II. [file 1471-2180-11-59-S1.PDF]

Additional File 1: Full list of genes differentially expressed in sorted *E. coli* cells

Full list of genes of *E. coli* differentially expressed in IMS sorted *E. coli* cells versus unsorted *E. coli* cells in two independent microarray studies I and II

Gene Name	Locus Tag	Fold-change of gene expression (sorted/unsorted*)		Annotation⊕
		Microarray study I	Microarray study II	
tldD	b3244	2.7 ± 1.4$^{\Psi}$	2.7 ± 1.4	Predicted peptidase
proW	b2678	2.4 ± 1.1	3.3 ± 1.3	Glycine betaine transporter subunit
ansP	b1453	2.2 ± 1.1	2.5 ± 1.1	*L*-asparagine transporter
ydhB	b1659	-2.2 ± 1.1	-2.9 ± 1.2	Predicted DNA-binding transcriptional regulator
yhhN	b3468	-2.6 ± 1.3	-3.1 ± 1.2	Conserved inner membrane protein
ygeV	b2869	-2.7 ± 1.1	-3.3 ± 1.4	Predicted DNA-binding transcriptional regulator
flhE	b1878	-2.7 ± 1.2	-3.2 ± 1.2	Conserved protein
yicG	b3646	-3.0 ± 1.2	-4.6 ± 1.3	Conserved inner membrane protein
ybbO	b0493	3.0± 1.5	/	*L*-asparagine permease
aslA	b3801	3.8 ± 1.2	/	(p)ppGpp synthetase I/GTP pyrophosphokinase)
yihO	b3876	/	6.3 ± 1.4$^{\Psi}$	Predicted transporter
ydaJ	b1338	/	4.2 ± 1.4	Predicted peptidase
mhpT	b0353	/	4.0 ± 1.5	Predicted 3-hydroxyphenylpropionic transporter
metL	b3940	/	3.9 ± 1.5	Fused aspartokinase II
ldcA	b1192	/	3.6 ± 1.7	*L, D*-carboxypeptidase A
yciT	b1284	/	3.6 ± 1.3	Predicted DNA-binding transcriptional regulator
ytfB	b4206	/	3.4 ± 1.2	Predicted cell envelope opacity-associated protein
lpxD	b0179	/	3.4 ± 1.3	UDP-3-O-(3-hydroxymyristoyl)-glucosamine N-acyltransferase
rbsD	b3748	/	3.3 ± 1.2	Glycine betaine transporter subunit
arsC	b3503	/	3.1 ± 1.3	Predicted cytoplasmic sugar-binding protein
recC	b2822	/	3.1 ± 1.4	Exonuclease V

ydaK	b1339	/	3.0 ± 1.4	Putative transcriptional regulator
pabA	b3360	/	2.9 ± 1.5	Arsenate reductase
ilvM	b3769	/	2.9 ± 1.6	Predicted peptidase
gpmA	b0755	/	2.8 ± 1.4	Acetolactate synthase II, small subunit
relA	b2784	/	2.7 ± 1.5	Aminodeoxychorismate synthase, subunit II
yciQ	b1268	/	2.7 ± 1.2	Phosphoglyceromutase I
uxaC	b3092	/	2.7 ± 1.4	DNA-binding transcriptional regulator
hdfR	b4480	/	2.6 ± 1.3	Conserved protein
ynfH	b1590	/	2.5 ± 1.3	Uronate isomerase
flgF	b1077	/	2.4 ± 1.3	Acrylsulfatase-like enzyme
yfaE	b2236	/	-2.3 ± 1.2	Oxidoreductase, membrane subunit
ybhG	b0795	/	-2.3 ± 1.4	Flagellar basal body rod protein
actP	b4067	/	-2.4 ± 1.1	Predicted oxidoreductase
ybcN	b0547	/	-2.4 ± 1.1	Predicted protein
flgD	b1075	/	-2.6 ± 1.4	Conserved inner membrane protein
ybaW	b0443	/	-2.7 ± 1.3	Putative transporter subunit
pckA	b3403	/	-2.7 ± 1.2	Part of gsp divergon involved in type II protein secretion
fliM	b1945	/	-2.8 ± 1.5	Fused glutathionylspermidine synthetase/amidase
hyfA	b2481	/	-2.9 ± 1.2	Predicted fimbrial-like adhesin protein
narH	b1225	/	-2.9 ± 1.3	Conserved protein
yhbM	b3163	/	-3.0 ± 1.4	Predicted DNA-binding transcriptional regulator
yadL	b0137	/	-3.6 ± 1.4	Conserved inner membrane protein
gsp	b2988	/	-3.7 ± 1.1	Nitrate reductase I, beta subunit
gspB	b3322	/	-4.1 ± 1.4	Conserved protein
yehY	b2130	/	-4.2 ± 1.2	Predicted DNA-binding transcriptional regulator
ycgK	b1178	/	-6.4 ± 1.1	Flagellar motor switch protein

*Sorted *E. coli* cells: *E. coli* cells treated with dispersion/homogenization and IMS cell sorting after pre-stored in RNA*later*; Unsorted *E. coli* cells: *E. coli* cells continuously stored in

RNA*later* without any treatment.

⊕Annotations are from NCBI Entrenz Gene Database, mostly limit to *E. coli* K-12 MG1655.

ΨMean ± geometric standard deviation from six replicates (two technical replicated slides × three built-in replicates per slide) for each gene; positive value indicates up-regulation in dispersed and IMS sorted cells, while negative value indicates down-regulation in dispersed and IMS sorted cells. Geometric standard deviation $=2^{SD}$, where SD is standard deviation of $\log_2$ transformation of fold-change.

/: not differentially expressed.
