# Supplementary material for: Separation of the bacterial species, Escherichia coli, from mixed-species microbial communities for transcriptome analysis
Source: BMC Microbiol. 2011 Mar 22;11:59. doi: 10.1186/1471-2180-11-59 (PMC3076228; doi:10.1186/1471-2180-11-59)
Supplement: Additional file 2 — qPCR primers for nine tested genes. List of primers and their optimized annealing temperatures used in qPCR to confirm differential expression in IMS sorted versus unsorted E. coli cells. [file 1471-2180-11-59-S2.PDF]

Additional File 2: qPCR primers for nine tested genes

List of primers and their optimized annealing temperatures used in qPCR to confirm differential expression in IMS sorted versus unsorted *E. coli* cells. The primers were designed using the software Primer Premier (Palo Alto, CA) and synthesized by Integrated DNA Technologies (Coralville, IA).

Gene	Primers (5'-3')	Annealing Temperature	Source
tldD	CTGACCGCCACCAAAGTTCG<br>GTGAATCCTACGCCCATCTG	52°C	this study
proW	ACTGAAGGGATCGACTGGG<br>GCTGGAAACCGTTGAGGA	52°C	this study
ansP	TGGCGCAGTGCTGTGAATTT<br>TCCTTGTGCTGATGGCGTTC	56°C	this study
ydhB	TACAGGTTGCCGATGATTC<br>GCTAAGCTGGAGTTGCGT	52°C	this study
yhhN	TGGCAGGTGAACTGTGGT<br>GAGCGGACGATCAGGAAG	56°C	this study
ygeV	CAATGCCAGGATAAACCG<br>CCGATAACTCTGCCACCG	50°C	this study
flhE	GCTACCAGCGTCATCAATC<br>TGTGGCAGGCAAGTAGTGT	50°C	this study
yicG	GAGTTGTATGCCGGTGTCT<br>TGTCCTTCGTGGCTGTAGT	54°C	this study
16S RNA	Eu338 (ACTCCTACGGGAGGCAGCAG)<br>Eu518 (ATTACCGCGGCTGCTGG)	62°C	Einen et al. [1]

1. Einen J, Thorseth IH, Ovreas L: **Enumeration of Archaea and Bacteria in seafloor basalt using real-time quantitative PCR and fluorescence microscopy**. *FEMS Microbiol Lett* 2008, **282**(2):182-187.
